# Soluble guanylate cyclase redox state under oxidative stress conditions in isolated monkey coronary arteries

**DOI:** 10.1002/prp2.261

**Published:** 2016-09-16

**Authors:** Masashi Tawa, Tomio Okamura

**Affiliations:** ^1^ Department of PharmacologyShiga University of Medical ScienceOtsuShigaJapan

**Keywords:** Coronary artery, oxidative stress, redox state, sGC activator, sGC stimulator, soluble guanylate cyclase

## Abstract

Coronary artery disease is associated with oxidative stress due to the excessive generation of free radicals in the vascular wall. This study investigated the impact of tert‐butyl hydroperoxide (t‐BuOOH), a peroxyl radical generator, on the redox state of soluble guanylate cyclase (sGC) in isolated monkey coronary arteries. Helically cut strips of endothelium‐intact monkey coronary arteries treated with the nitric oxide synthase inhibitor N^G^‐nitro‐L‐arginine (10 *μ*mol/L) were exposed for approximately 60 min to either no drug or t‐BuOOH (100 *μ*mol/L) in the presence and absence of *α*‐tocopherol (300 *μ*mol/L). Relaxation and cGMP levels in response to the sGC stimulator BAY 41‐2272 and the sGC activator BAY 60‐2770 were assessed by organ chamber technique and enzyme immunoassay, respectively. The relaxant response to BAY 41‐2272 was significantly impaired by the exposure to t‐BuOOH, whereas the response to BAY 60‐2770 was significantly augmented. In addition, vascular cGMP accumulation caused by BAY 41‐2272 was decreased by the exposure to t‐BuOOH, whereas for BAY 60‐2770, it was increased. These effects of t‐BuOOH were abolished by coincubation with *α*‐tocopherol. Furthermore, correlations were observed between BAY compound‐induced relaxant magnitudes and cGMP levels. Therefore, it is concluded that increased oxidative stress leads to disruption of the sGC redox state in monkey coronary arteries. This finding is of great importance for understanding coronary physiology in primates.

**Abbreviations**
ANOVA: analysis of variance
CAD: coronary artery disease
E_max_: maximal response
L‐NA: N^G^‐nitro‐L‐arginine
NO: nitric oxide
pD_2_: negative logarithm of the dilator concentration that caused half the E_max_
PG: prostaglandin
sGC: soluble guanylate cyclase
t‐BuOOH: tert‐butyl hydroperoxide

## Introduction

Coronary artery disease (CAD), a major cause of death, is characterized by a reduction in coronary blood flow due to spasm, thrombus, embolus, and stenosis (Buja 2015). Excessive oxidative stress caused by free radicals in the primary lesion is an important feature of CAD. For example, the most reactive‐free radical superoxide is increased in ischemic/reperfused canine coronary arteries (Mykytenko et al. 2007), in atherosclerotic coronary arteries from high cholesterol diet‐fed monkeys (Supari et al. 1995), and in narrowed porcine coronary arteries after balloon injury (Nunes et al. 1997). An increase in superoxide production was also confirmed in the coronary arteries of patients with CAD (Azumi et al. 2002; Sorescu et al. 2002; Guzik et al. 2006). Moreover, the oxidative damage of cell membranes caused by free radicals is linked to human coronary atherogenesis (Mehrabi et al. 1999; van Dijk et al. 2012).

The nitric oxide (NO)/soluble guanylate cyclase (sGC)/cGMP pathway has pivotal roles, such as vasodilation, antiaggregation, anti‐inflammation, and antiproliferation, in the coronary circulation system (Toda et al. 2011). Although NO stimulates cGMP synthesis by activating sGC, this cytosolic enzyme exists in either the NO‐sensitive reduced form or the NO‐insensitive oxidized/heme‐free form. Briefly, if the ferrous (Fe^2+^) heme moiety is oxidized to the ferric (Fe^3+^) state or lost, NO loses its ability to activate sGC (Evgenov et al. 2006; Stasch et al. 2011). In recent years, it has been proposed that a shift of the sGC redox equilibrium toward the NO‐insensitive state contributes to the onset and development of some cardiovascular diseases (Stasch et al. 2006; Chester et al. 2011). In contrast, sGC in coronary arteries may also be subjected to such redox regulation (Tawa et al. 2014a).

One determinant of vascular sGC redox equilibrium is the type of free radical and its derivatives. Superoxide, peroxynitrite, and hydrogen peroxide was suggested to be able to change sGC from the reduced form to the oxidized/heme‐free form in isolated rat aortas (Stasch et al. 2006) and iliac arteries (Tawa et al. 2014b, 2015a) or in cultured sheep pulmonary artery smooth muscle cells (Zhou et al. 2008). However, there is no firm evidence that the sGC redox state in the coronary artery is also affected under oxidative stress conditions. Therefore, this study investigated this effect in isolated monkey coronary arteries by analyzing the influence of the peroxyl radical generator tert‐butyl hydroperoxide (t‐BuOOH). As now known, lipid peroxyl radicals are mediators of coronary atherogenesis.

## Materials and Methods

### Animal

Three male (14 to 15 years old) and three female (11 to 12 years old) crab‐eating monkeys (*Macaca irus*) weighing 4 to 7 kg were used for this study. These monkeys had no cardiovascular or metabolic diseases. All procedures involving animals were approved by the Animal Care and Use Committee at Shiga University of Medical Science, and this study was performed based on guidelines in accordance with the recommendations of the Weatherall Report on “The Use of Non‐Human Primates in Research”. The monkeys were housed individually in cages with various toys for environmental enrichment under controlled conditions of humidity (50 ± 5%), temperature (25 ± 2°C), and light (12 h light/12 h dark cycle), and their health and welfare were ensured by the researchers and the animal care staff with daily monitoring. A commercial primate pellet diet and fresh fruit/vegetables were given daily, and drinking water was available ad libitum. Routine anesthetic and/or analgesic agents were used appropriately, and every effort was made to minimize animal discomfort and pain.

### Preparation

Each monkey under deep general anesthesia with ketamine (10 mg/kg, i.m.) and sodium pentobarbital (40 mg/kg, i.v.) was killed by bleeding, and the heart was rapidly excised. Coronary arteries (0.5 to 1.0 mm outside diameter) were isolated, the surrounding tissues cleaned, and the arteries were then cut helically into strips, with special care taken to preserve the endothelium. The functional integrity of the endothelium was verified by observing a relaxant response to acetylcholine (1 *μ*mol/L). The strips were fixed vertically between hooks in a muscle bath (10 mL capacity) containing modified Ringer‐Locke solution, which was aerated with a gas mixture 95% O_2_ and 5% CO_2_. The pH and temperature were maintained throughout the experiment at 7.4 and 37 ± 0.3°C, respectively. Constituents of the modified Ringer‐Locke solution were as follows (mmol/L): NaCl 120, KCl 5.4, CaCl_2_ 2.2, MgCl_2_ 1.0, NaHCO_3_ 25.0, and glucose 5.6. The hook anchoring the upper end of the strip was connected to the lever of an isometric force‐displacement transducer (Nihon Kohden Kogyo Co., Tokyo, Japan). The resting tension of the strip was adjusted to 1.0 g, which is optimal for inducing maximum contraction. Before the start of experiments, all strips were allowed to equilibrate in the bathing medium for 60 to 90 min. During this time the solution was replaced every 10 to 15 min.

### Mechanical responses

Isometric contractions and relaxations were displayed on an ink‐writing oscillograph. Because the presence of endothelium‐derived NO makes it difficult to assess the sGC redox state (Tawa et al. 2015b), the following experiments were performed in the presence of the NO synthase inhibitor N^G^‐nitro‐L‐arginine (L‐NA, 10 *μ*mol/L). The preparations were exposed to no drug for 60 min (control) or to t‐BuOOH (100 *μ*mol/L) with and without *α*‐tocopherol (300 *μ*mol/L) in the bathing solution. These concentrations were determined based on previous studies (de Nigris et al. 2000; Stevanovic et al. 2009). The strips were then treated with prostaglandin (PG) F_2*α*_ in a concentration range 1 to 3 *μ*mol/L to obtain an adequate precontraction level, which was not significantly different between groups [developed tension, 0.46 ± 0.05 g (control), 0.47 ± 0.07 g (t‐BuOOH), and 0.58 ± 0.06 g (+*α*‐tocopherol)]. After the contraction reached a plateau, concentration–response curves for the reduced sGC stimulant BAY 41‐2272 and the oxidized/heme‐free sGC stimulant BAY 60‐2770 were obtained by adding the drug directly to the bathing medium in cumulative concentrations. At the end of each experiment, 100 *μ*mol/L papaverine was added to induce maximal relaxation, which was taken as 100% for the relaxations induced by agonists.

### cGMP measurements

The content of cGMP in monkey coronary artery strips was measured according to a method described previously (Tawa et al. 2014a). Briefly, the strips, which were precontracted with PGF_2*α*_ after exposure to each condition stated above, were treated with BAY 41‐2272 (100 nmol/L) or BAY 60‐2770 (1 nmol/L) for approximately 30 min and were then immediately plunged into liquid nitrogen. To evaluate the correlation between relaxation and cGMP accumulation, the vasorelaxant ability was calculated as a percentage of PGF_2*α*_‐induced precontraction. The tissues were homogenized in 300 *μ*L of 5% trichloroacetic acid at 0°C with a glass homogenizer. After centrifugation at 1500 *g* for 10 min, water‐saturated ether was added to the collected supernatant, and the residual ether was removed from the aqueous layer by heating the sample at 70°C for 5 min. An aliquot of extract was then used for cGMP determination, using a commercially available enzyme immunoassay kit (Cayman Chemical Co., Ann Arbor, MI). The cGMP level in the tissue was normalized to the protein content measured in the same extract. Protein content was determined by the Bradford assay.

### Drugs

The following drugs were used: t‐BuOOH, *α*‐tocopherol, and L‐NA (Sigma Chemical Co., St. Louis, MO); BAY 41‐2272 and BAY 60‐2770 (kindly provided by Dr. Johannes‐Peter Stasch of the Institute of Cardiovascular Research, Pharma Research Centre, Bayer AG, Wuppertal, Germany); PGF_2*α*_ (Pharmacia‐Upjohn, Tokyo, Japan); ketamine (Sankyo, Tokyo, Japan); sodium pentobarbital and papaverine hydrochloride (Dainippon‐Sumitomo Pharma Co., Osaka, Japan). Ethanol, dimethyl sulfoxide, and sodium bicarbonate buffer (pH 9.2) were used as a solvent for *α*‐tocopherol, BAY compounds, and PGF_2*α*_, respectively. Distilled water was used to dissolve all other drugs and to prepare serial dilutions, as required, from stocks on the day of the experiment.

### Statistics

All values are expressed as the mean ± SEM. Concentration–response curves were fitted by nonlinear regression analysis using the Graph Pad Prism 6.0 software (Graph Pad Software Inc., San Diego, CA), and the maximal response (E_max_) and negative logarithm of the dilator concentration that caused half the E_max_ (pD_2_) were determined. Concentration–response curves were analyzed with two‐way repeated measures analysis of variance (ANOVA) and Bonferroni post hoc test. E_max_ and pD_2_ values were compared using the Bonferroni multiple comparisons test after one‐way ANOVA. Comparison of the cGMP levels was performed using one‐way ANOVA followed by Bonferroni post test. Correlations were tested using the Pearson correlation test. Significant differences were considered significant at *P *<* *0.05.

## Results

### Influence of t‐BuOOH on BAY compound‐induced vasorelaxation

**Figure 1:**
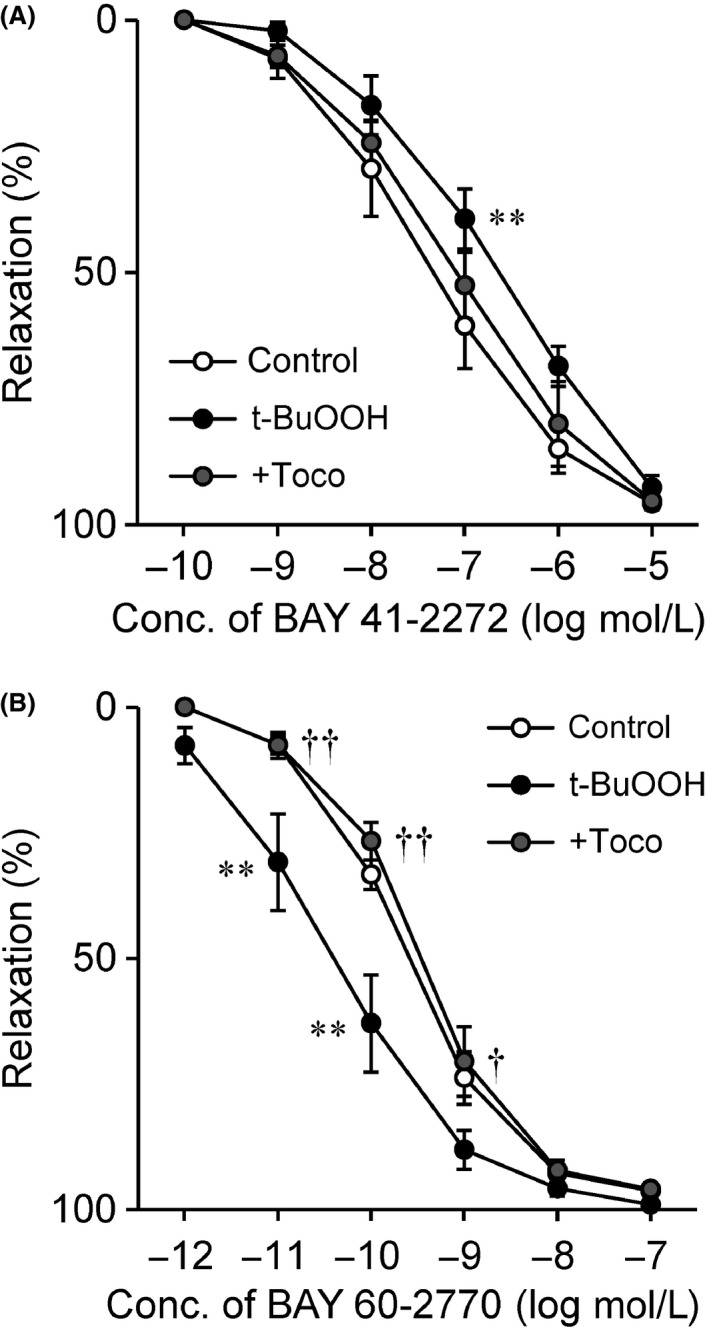
Effects of t‐BuOOH on BAY 41‐2272 (A)‐ and BAY 60‐2770 (B)‐induced relaxation of endothelium‐intact monkey coronary arteries treated with N^G^‐nitro‐L‐arginine in the absence or presence of *α*‐tocopherol. The relaxation is presented as a value relative to that induced by papaverine. White, control; black, t‐BuOOH; gray, +*α*‐tocopherol (Toco). Each point and bar represents the mean ± SEM of six experiments. **P <* *0.01, compared to the control; ^†^ *P *<* *0.05 and ^††^ *P *<* *0.01, compared to t‐BuOOH. Statistical analysis was performed using two‐way repeated measures ANOVA with Bonferroni post hoc test.

The addition of either BAY 41‐2272 at concentrations of 100 pmol/L to 10 *μ*mol/L or BAY 60‐2770 at 1 pmol/L to 100 nmol/L produced a concentration‐dependent relaxation of endothelium‐intact monkey coronary arteries treated with L‐NA in all experimental groups. The efficacies of these drugs, as reflected by the E_max_ values, were comparable between groups (Table 1). However, as shown in Figure 1A and B, t‐BuOOH exposure resulted in a rightward displacement of the concentration–response curve for BAY 41‐2272, whereas the BAY 60‐2770 concentration–response curve was shifted to the left. In addition, t‐BuOOH exposure somewhat decreased the pD_2_ values for BAY 41‐2272 but increased those for BAY 60‐2770 (Table 1), which indicated a reduced and enhanced sensitivity of the arteries to the former and the latter, respectively. These effects of t‐BuOOH were significantly inhibited in the presence of *α*‐tocopherol (Fig. 1A and B; Table 1).

**Table 1:** pD_2_ and E_max_ for BAY 41‐2272 and BAY 60‐2770

	Control	t‐BuOOH	+*α*‐tocopherol
BAY 41‐2272			
pD_2_	7.39 ± 0.28	6.89 ± 0.24	7.04 ± 0.27
E_max_ (%)	93.8 ± 1.5	89.0 ± 3.2	95.2 ± 1.8
BAY 60‐2770			
pD_2_	9.61 ± 0.11	10.40 ± 0.25^a^	9.46 ± 0.12^b^
E_max_ (%)	95.5 ± 1.0	96.4 ± 0.8	96.2 ± 1.5

Data are the mean ± SEM of six experiments.

a *P *<* *0.05, compared to the control.

b *P *<* *0.01, compared to t‐BuOOH. Statistical analysis was performed using one‐way ANOVA with Bonferroni post hoc test.

### Influence of t‐BuOOH on BAY compound‐induced cGMP accumulation

**Figure 2:**
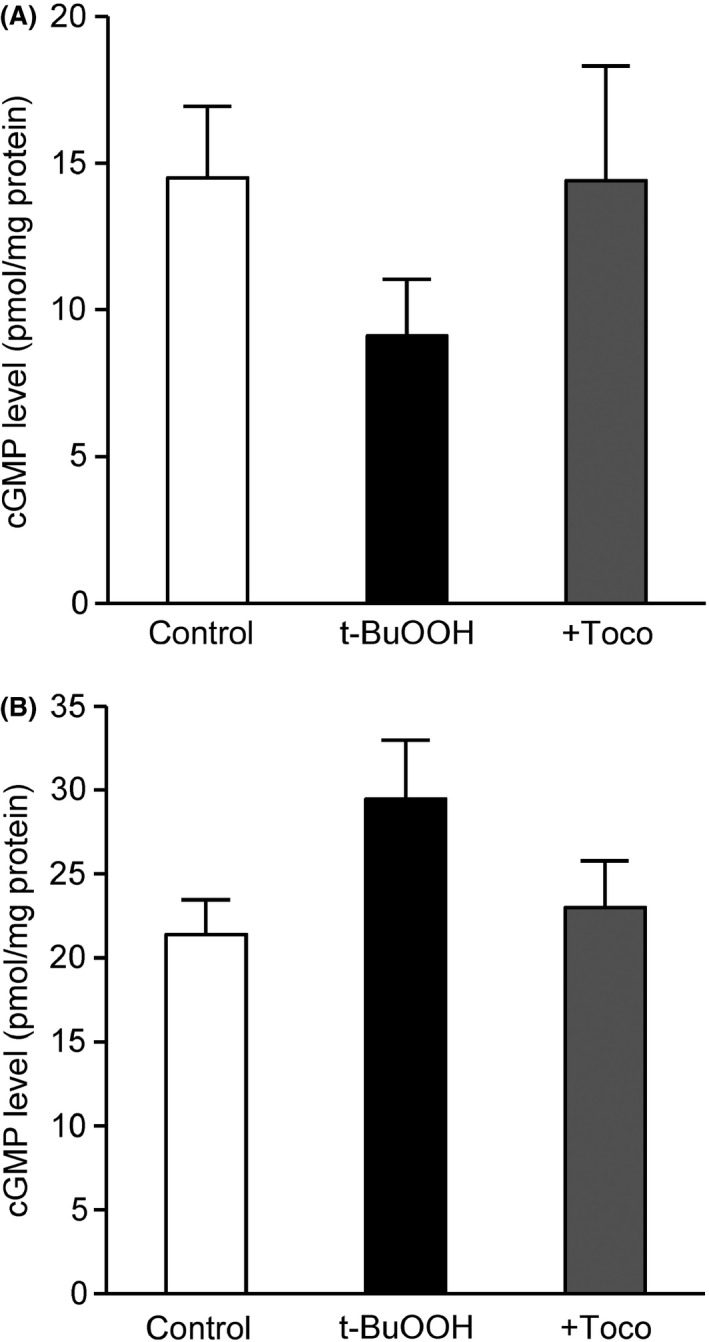
Effects of t‐BuOOH on BAY 41‐2272 (A)‐ and BAY 60‐2770 (B)‐induced cGMP accumulation in endothelium‐intact monkey coronary arteries treated with N^G^‐nitro‐L‐arginine in the absence or presence of *α*‐tocopherol. White, control; black, t‐BuOOH; gray, +*α*‐tocopherol (Toco). Each column and bar represents the mean ± SEM of six experiments. Statistical analysis was performed using one‐way ANOVA with Bonferroni post hoc test.

As shown in Figure 2A, cGMP levels in endothelium‐intact monkey coronary arteries stimulated with BAY 41‐2272 (100 nmol/L) were decreased by t‐BuOOH exposure, which was reversed by coincubation with *α*‐tocopherol. In contrast, Figure 2B shows that BAY 60‐2770 (1 nmol/L) induced greater cGMP accumulation in the presence of t‐BuOOH than in its absence. This augmentation by t‐BuOOH was not observed when *α*‐tocopherol was present.

### Correlation between relaxation and cGMP accumulation

**Figure 3:**
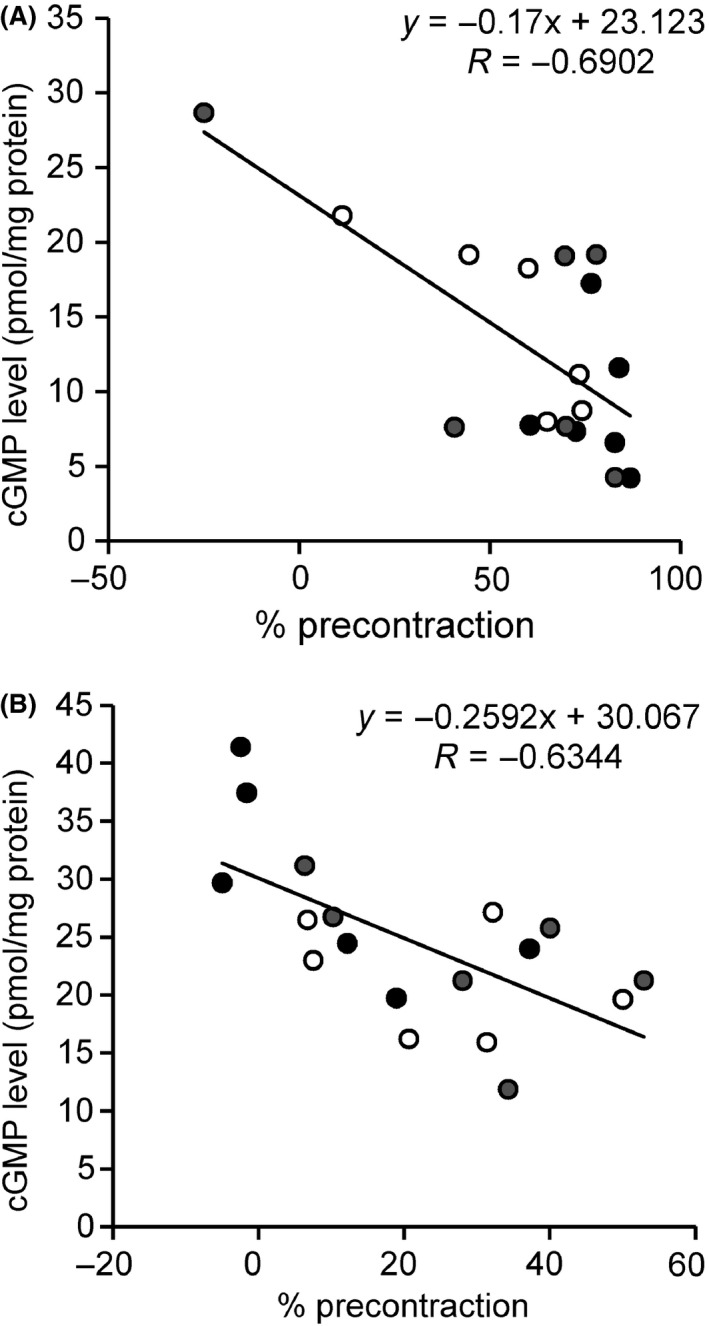
Relationship between relaxation and cGMP accumulation in response to BAY 41‐2272 (A) or BAY 60‐2770 (B). The relaxation ability was calculated as a percentage of precontraction induced by prostaglandin F_2*α*_ (% precontraction). White, control; black, t‐BuOOH; gray, +*α*‐tocopherol. Statistical analysis was performed using Pearson correlation test.

In endothelium‐intact monkey coronary arteries treated with L‐NA, the degrees of remaining contraction (% precontraction) after BAY 41‐2272 addition showed a negative relationship (*P *=* *0.0015) with the tissue cGMP levels (Fig. 3A), which indicated an association between this compound‐induced relaxation and cGMP synthesis. Similar to BAY 41‐2272, there was also a good correlation (*P *=* *0.0047) for BAY 60‐2770 to cause relaxation and stimulate cGMP accumulation (Fig. 3B). These results suggest that the changes in relaxant responses to BAY compounds by exposure to t‐BuOOH are attributable to an increase or decrease in cGMP production.

## Discussion

The NO/sGC/cGMP pathway is often abnormal in the diseased coronary artery (Förstermann et al. 1988; Berkenboom et al. 1989; Schächinger and Zeiher 1995; Yamagishi et al. 1995). In this regard, the sGC redox equilibrium is directly linked to NO bioavailability and attention should be paid to its regulatory system. Therefore, the objective of this study was to evaluate the mechanism by which the redox state of coronary sGC is controlled under oxidative stress conditions. Additionally, responses to BAY 41‐2272, a reduced sGC stimulant, and BAY 60‐2770, an oxidized/heme‐free sGC stimulant, were examined to achieve this goal. The BAY 41‐2272‐evoked relaxation of monkey coronary arteries was impaired under stress conditions caused by t‐BuOOH, which was coupled with decreased cGMP generation. In contrast, BAY 60‐2770 produced greater relaxation as well as higher levels of cGMP in coronary arteries exposed to t‐BuOOH. Consistent with these results, Safaya et al. (2005) showed that exogenously applied lysophosphatidylcholine, a lipid peroxidation product, attenuates the relaxation of porcine coronary arteries induced by an NO donor through the induction of oxidative stress. Taken together, these findings suggest that the heme iron of sGC in the coronary artery undergoes oxidation under oxidative stress conditions, resulting in a shift of the redox balance toward the NO‐insensitive state. This is the first evidence to show a relationship between oxidative stress and the sGC redox state in the coronary artery and is important for understanding coronary atherosclerosis. However, it is necessary to examine the sGC redox state in atherosclerotic coronary arteries in the future.

t‐BuOOH generates peroxyl radicals by stimulating lipid peroxidation, which can lead to oxidative stress conditions (Ayala et al. 2014). Because the peroxyl radical scavenger *α*‐tocopherol prevented the t‐BuOOH‐induced alterations in vascular reactivity and cGMP accumulation in response to either BAY 41‐2272 or BAY 60‐2770, it is likely that peroxyl radicals contribute to disruption of the sGC redox homeostasis in the coronary artery. This is supported by other studies showing the heme iron‐oxidizing potential of peroxyl radicals in heme‐containing proteins other than sGC (Nantes et al. 2000; Goldstein and Samuni 2005). However, a variety of pro‐oxidant molecules are produced during the lipid peroxidation process (Ayala et al. 2014); therefore, it is also possible that this radical species is not a direct contributor to the sGC redox shift and is only an inducer. Regardless, because lipid peroxidation is a fundamental process in coronary atherogenesis (Stocker and Keaney 2004), it is not surprising that the change in sGC from the NO‐sensitive reduced form to the NO‐insensitive oxidized/heme‐free form occurs in the coronary artery in vivo. In fact, the vasodilator response to intracoronary‐injected nitroglycerin, an NO‐donating drug, is impaired in atherosclerotic plaque lesions (Schächinger and Zeiher 1995; Yamagishi et al. 1995). Future studies regarding the effectiveness of intracoronary injection of an sGC stimulator and an sGC activator will further support the above conclusion.

*α*‐tocopherol, a natural form of vitamin E, was used as a tool for elucidating the mechanism of t‐BuOOH‐induced actions in this study. Briefly, the results obtained may not always indicate the utility of *α*‐tocopherol as a therapeutic agent. Verlangieri and Bush (1992) histologically demonstrated the antiatherogenic effect of dietary *α*‐tocopherol in monkeys, whereas Rainwater et al. (2007) reported that vitamin E supplementation induces smaller high‐density lipoprotein size in baboons. In addition, the results of clinical trials using vitamin E for the treatment of CAD have been inconsistent (Kirmizis and Chatzidimitriou 2009). Because several studies assessing the usefulness of vitamin E are continuously in progress, the answer of whether *α*‐tocopherol is of clinical benefit will be obtained in due course.

Several studies demonstrated that sex differences are present in coronary function (Cox and Cohen 1997; Barber et al. 1998; Wong et al. 2015). In this regard, although a small sample number and mismatched age are not suitable for making a conclusion, similar influences of t‐BuOOH on vascular reactivity to BAY compound were observed in coronary arteries obtained from male (Fig. S1) and female (Fig. S2) monkeys. However, the effects in females were greater than in males. This may be because male coronary arteries, compared to female coronary arteries, are intrinsically under high oxidative stress conditions (Wong et al. 2015). Age mismatch may not be a cause of the sex difference in t‐BuOOH‐induced effects because our recent study showed that aging does not affect vascular sGC redox equilibrium (Shimosato et al. 2016). Anyway, it is likely that if excessive oxidative stress occurs in the coronary artery, the sGC redox equilibrium shifts to the NO‐insensitive state regardless of sex.

There is currently no analytical method to quantitate the expression of individual forms of sGC at the tissue level. Therefore, according to a number of previous reports (Zhou et al. 2008; Costell et al. 2012; Tawa et al. 2014a,b, 2015a,b; Goulopoulou et al. 2015), two different NO‐independent stimulants acting on sGC were used in this study as valuable tools for indirectly assessing the redox state of the enzyme. As described elsewhere (Evgenov et al. 2006; Stasch et al. 2011), NO acts synergistically with the sGC stimulator BAY 41‐2272, but only additively with the sGC activator BAY 60‐2770. Although this is the reason why this study was performed in the presence of L‐NA, whether this condition is physiological is unclear. Of course, coronary endothelial function is impaired in patients with CAD (Gutiérrez et al. 2013), yet it must be multifarious how much the endothelium‐derived NO production is decreased. Because there is a report showing that NO reacts with and traps peroxyl radicals (O'Donnell et al. 1997), the outcomes might differ in the presence and absence of L‐NA. Even so, the conclusion that oxidative stress interferes with the sGC redox equilibrium in the coronary artery does not change.

Another limitation of this study is that the influence of chronic exposure to t‐BuOOH was not assessed. Oxidation of the sGC heme moiety from the ferrous to ferric state subsequently results in heme detachment from the enzyme, which facilitates degradation of the enzyme by the ubiquitin‐proteasome system (Meurer et al. 2009). In fact, Neo et al. (2011) indicated an sGC depleting effect of the heme specific oxidant ODQ in organoid cultured bovine coronary arteries. Furthermore, the same group showed that prolonged exposure of coronary arteries to angiotensin II causes the downregulation of sGC accompanied by oxidative stress induction (Patel et al. 2016). Consequently, the efficacy of sGC activators as well as NO donors and sGC stimulators may be reduced under chronic oxidative stress conditions. This is a future research issue.

In conclusion, this study demonstrated that t‐BuOOH‐induced oxidative stress conditions lead to a shift of the sGC redox equilibrium toward the oxidized/heme‐free form in monkey coronary arteries. This finding will provide a better understanding of the mechanism by which the NO/sGC/cGMP pathway is regulated in the diseased coronary artery in primates.

## Author Contributions

Tawa and Okamura participated in research design, wrote or contributed to the writing of the manuscript. Tawa also conducted the experiments and performed data analysis.

## Disclosures

None declared.

## Supporting information


**Figure S1.** Effects of t‐BuOOH on BAY 41‐2272 (A)‐ and BAY 60‐2770 (B)‐induced relaxation of endothelium‐intact male monkey coronary arteries treated with N^G^‐nitro‐L‐arginine in the absence or presence of *α*‐tocopherol. The relaxation is presented as a value relative to that induced by papaverine. White, control; black, t‐BuOOH; gray, +*α*‐tocopherol (Toco). Each point and bar represents the mean ± SEM of three experiments. *P <* *0.05, compared to the control; ^††^ *P *<* *0.01, compared to t‐BuOOH. Statistical analysis was performed using two‐way repeated measures ANOVA with Bonferroni post hoc test.

**Figure S2.** Effects of t‐BuOOH on BAY 41‐2272 (A)‐ and BAY 60‐2770 (B)‐induced relaxation of endothelium‐intact female monkey coronary arteries treated with N^G^‐nitro‐L‐arginine in the absence or presence of *α*‐tocopherol. The relaxation is presented as a value relative to that induced by papaverine. White, control; black, t‐BuOOH; gray, +*α*‐tocopherol (Toco). Each point and bar represents the mean ± SEM of three experiments. *P <* *0.05 and **P <* *0.01, compared to the control; ^††^ *P *<* *0.01, compared to t‐BuOOH. Statistical analysis was performed using two‐way repeated measures ANOVA with Bonferroni post hoc test.
