# Supplementary figures and images for: Soluble guanylate cyclase redox state under oxidative stress conditions in isolated monkey coronary arteries
Source: Pharmacol Res Perspect. 2016 Sep 16;4(5):e00261. doi: 10.1002/prp2.261 (PMC5045941; doi:10.1002/prp2.261)

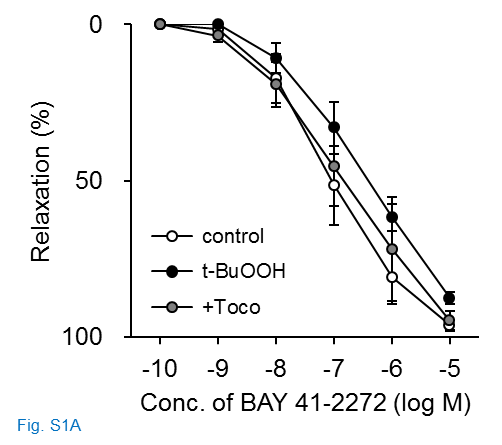

Supplement: Supplementary file 1 — Figure S1. Effects of t‐BuOOH on BAY 41‐2272 (A)‐ and BAY 60‐2770 (B)‐induced relaxation of endothelium‐intact male monkey coronary arteries treated with NG‐nitro‐L‐arginine in the absence or presence of α‐tocopherol. The relaxation is presented as a value relative to that induced by papaverine. White, control; black, t‐BuOOH; gray, +α‐tocopherol (Toco). Each point and bar represents the mean ± SEM of three experiments. *P < 0.05, compared to the control; †† P < 0.01, compared to t‐BuOOH. Statistical analysis was performed using two‐way repeated measures ANOVA with Bonferroni post hoc test. [file PRP2-4-e00261-s001.tif]

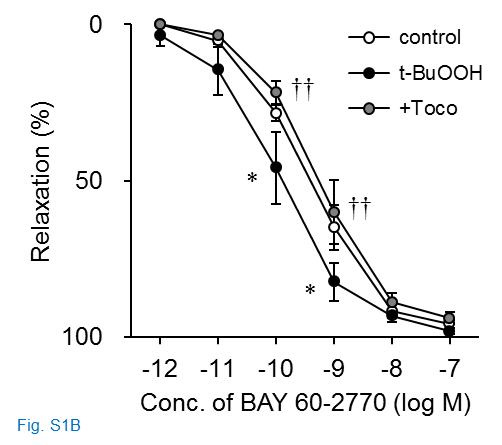

Supplement: Supplementary file 2 [file PRP2-4-e00261-s002.tif]

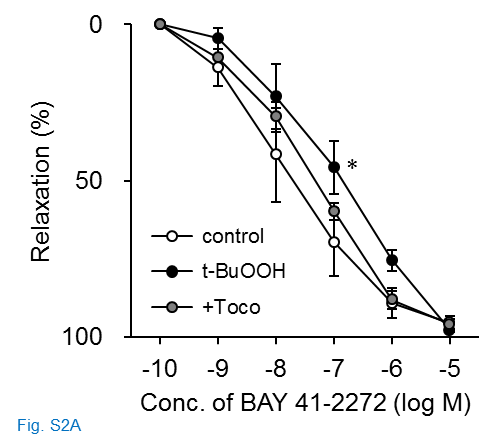

Supplement: Supplementary file 3 — Figure S2. Effects of t‐BuOOH on BAY 41‐2272 (A)‐ and BAY 60‐2770 (B)‐induced relaxation of endothelium‐intact female monkey coronary arteries treated with NG‐nitro‐L‐arginine in the absence or presence of α‐tocopherol. The relaxation is presented as a value relative to that induced by papaverine. White, control; black, t‐BuOOH; gray, +α‐tocopherol (Toco). Each point and bar represents the mean ± SEM of three experiments. *P < 0.05 and **P < 0.01, compared to the control; †† P < 0.01, compared to t‐BuOOH. Statistical analysis was performed using two‐way repeated measures ANOVA with Bonferroni post hoc test. [file PRP2-4-e00261-s003.tif]

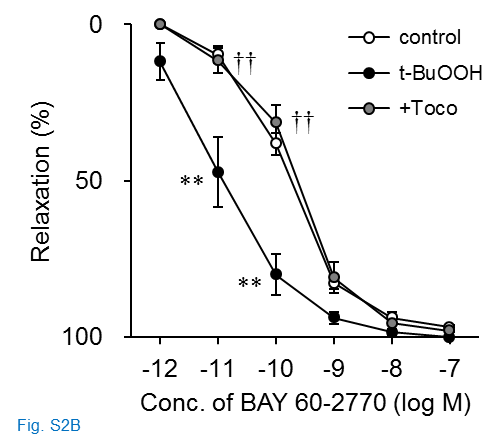

Supplement: Supplementary file 4 [file PRP2-4-e00261-s004.tif]
